# Editorial: Host immune evasion by *Mycobacterium tuberculosis*: Current updates

**DOI:** 10.3389/fimmu.2022.1102415

**Published:** 2022-12-13

**Authors:** Meseret Habtamu, Adane Miheret, Anne Spurkland

**Affiliations:** ^1^Department of Mycobacteria diseases Research, Armauer Hansen Research Institute (AHRI), Addis Ababa, Ethiopia; ^2^Department of Bacterial and Viral Diseases Research, Armauer Hansen Research Institute (AHRI), Addis Ababa, Ethiopia; ^3^Department of Molecular Medicine, Institute of Basic Medical Sciences, Faculty of Medicine, University of Oslo, Oslo, Norway

**Keywords:** immune evasion, PPAR γ, foamy macrophage, arabinogalactan, innate immunity, *M.tb*

*Mycobacterium tuberculosis (M. tb)* can become a long-term infection by evading the host’s immune response. Virulent mycobacterial strains employ various strategies to hijack the host’s immune systems in favor of persistent infection and disease progression. Although the immune evasion mechanisms identified so far are already numerous, they most likely represent only the tip of the iceberg. Identifying fundamental pathogen virulence regulators and investigating the underlying immune mechanisms remain critical in the quest for more effective vaccines and therapeutic targets.

Ubiquitin (Ub) targeting intracellular bacteria is a crucial innate immune mechanism in mammalian cells against intracellular pathogens. Herpesvirus-associated ubiquitin-specific protease (HAUSP USP7) is a deubiquitinase (DUB) that mediates the expression and function of proteins regulating cellular processes and modulating their state of ubiquitination (1, 2). Kim et al., 2022, now add this protease to the list of proteins exploited by *M. tb* to avoid host immune responses.

*M. tb* possesses a unique family of proteins, named PE_PGRS. Recently, an increasing number of reports have shown that the mycobacterial PE_PGRS proteins play critical roles in bacterial pathogenesis and immune evasion (3). PE_PGRS38, encoded by Rv2162c in pathogenic mycobacterial species (4), is identified as a potential target for the second-line anti-TB drug, Capreomycin (5). Kim et al., 2022, found that PE_PGRS38 binds to HAUSP and thereby regulates the activity of various proteins through modulation of their state of ubiquitination. The overall downstream effect of PE_PGRS38 interaction with HAUSP increased intracellular survival of the bacteria and downregulated inflammatory cytokine levels.

TNF Receptor Associated Factor 6 (TRAF6) is an intermediate protein in inflammatory-related signaling pathways and the regulation of its cytosolic level is crucial in maintaining cellular homeostasis (6–8). Mycobacterial antigens have been reported to interact with TRAF6 and thus regulate inflammation in the host. HAUSP is a positive regulator of TRAF6 in macrophages, allowing increased stability following lipopolysaccharide treatment. On the other hand, the interaction between HAUSP and PE_PGRS38 promotes the degradation of TRAF6 by inducing ubiquitination, which results in alterations in cytokine levels and increased bacterial persistence as a downstream effect (Kim et al., 2022). Taken together, PE_PGRS38 could be a potential virulence factor for *M. tb* involved in modulating cytokine production by directly binding to HAUSP *via* regulation of TRAF6.

A complex cell-wall structure acts as a natural barrier between the slow-growing mycobacterium species and the uptake of antibiotics. The highly branched arabinogalactan (AG), and the characteristic long-chain mycolic acid, forms the mycobacterial cell-wall core (9). *EmbA* and *EmbB* are among the key genes involved in AG synthesis (10). The EmbA–EmbB–AcpM2 complex is the target for the anti-TB drug, ethambutol, where over-expression of *embA* and *embB* is associated with high-level ethambutol resistance (11–14). Li et al., 2022 showed a reduced level of ethambutol resistance in the EmbA-KD strain, which causes early clearance of *M. marinum* by the host innate immunity through reprogramming of the oxidative metabolism of macrophages, suggesting potential targets for anti-TB immune intervention.

Foamy macrophages are associated with chronic inflammation in metabolic, infectious, or autoimmune diseases. *M. tb*-infected foamy macrophages (FMs) represent a hallmark of TB lesions and are characterized by bubble-like lipid bodies in their cytoplasm (15) with morphologic and functional alterations including reduced phagocytic and antimicrobial activity (16). FMs play an important role in the development of active TB diseases and infection dissemination. This is governed *via* modulating nuclear transcription receptors including the lipid-activated nuclear receptor, such as peroxisome proliferator-activated receptor gamma (PPARgamma) as well as membrane receptors such as CD36, SR-A1, and ABCG1, *via* enhanced lipid intake and cholesterol efflux (17).

The relative lipid composition of FMs differ in disease contexts, which could be attributed to the expression and function of PPARgamma by regulating intake/efflux. In silicosis, PPARgamma antagonist GW9662 treatment downregulated CD36 expression and inhibited FM formation (18). In alveolar proteinosis, PPARgamma regulates the formation of FM by modulating the expression of ABCG1 (19).

A study by Ye et al., 2022, investigated the molecular mechanisms involved and the role of PPARgamma activation on the expression of lipid metabolism-related molecules. The results showed that reduced PPARgamma expression was accompanied by increased FM formation. Using THP-1 cells, the authors provided evidence that PPARgamma plays an important role in regulating lipid metabolism in TB-related FM formation.

Together, the studies included in this thematic ‘Research Topic’ suggest fundamental mechanisms for how mycobacterial virulence factors circumvent various aspects of the host immune function to promote *M. tb* pathogenesis. These molecular pathways may represent novel or improved targets for vaccines and/or anti-TB drugs.

## Author contributions

MH designed and prepared the original editorial draft. AM and AS revised the manuscript. All authors contributed to the article and approved the submitted version.
